# Perspectives Toward Long-Term Care Measurement From Frontline Workers in Brazil

**DOI:** 10.1093/geroni/igab046.611

**Published:** 2021-12-17

**Authors:** Ruth Caldeira de Mello, Patrick Wachholz, Paulo José Fortes Villas Boas, Vivian Schutz, Michael Lepore, Deanna Myer, Ester Villalonga Olives, Kirsten Corazzini

**Affiliations:** 1 University of São Paulo, São Paulo, Sao Paulo, Brazil; 2 São Paulo State University, São Paulo State University - Unesp, Sao Paulo, Brazil; 3 University of São Paulo, Universidade de São Paulo, Sao Paulo, Brazil; 4 University of Maryland Baltimore, School of Nursing, Baltimore, Maryland, United States; 5 LiveWell Alliance, Southington, Connecticut, United States; 6 University of Maryland School of Nursing, Baltimore, Maryland, United States; 7 University of Maryland, School of Pharmacy, Baltimore, Maryland, United States

## Abstract

The Brazilian long-term care (LTC) sector remains poorly structured and underdeveloped. COVID-19 did not bring unprecedented focus to the sector just because of the high mortality; it also affected the quality of care. In this pilot study, we evaluated the perspectives toward WE-THRIVE LTC measurements from Brazilian frontline workers in five long-term care facilities. For the four WE-THRIVE domains of LTC measurement (workforce and staffing, person-centered care, organizational context, and care outcomes), respondents used a 4-point Likert scale to rate their importance and answered open-ended questions about how these aspects of care changed since COVID-19. With few exceptions, respondents rated these aspects of LTC as extremely important or very important. Qualitative results highlighted concerns about and impacts of COVID-19, such as challenges related to the isolation of residents. The assessed measurement domains are confirmed to be important by frontline staff in Brazil. Measurement adoption must account for current issues.

